# Dexamethasone as an Analgesic Adjunct for Postcesarean Delivery Pain: A Randomized Controlled Trial

**DOI:** 10.1155/2021/4750149

**Published:** 2021-09-24

**Authors:** Jennifer E. Mehdiratta, Jennifer E. Dominguez, Yi-Ju Li, Remie Saab, Ashraf S. Habib, Terrence K. Allen

**Affiliations:** ^1^Department of Anesthesiology, Duke University Medical Center, Durham, NC 27710, USA; ^2^Department of Biostatistics and Bioinformatics, Duke University Medical Center, Durham, NC 27710, USA; ^3^Duke Molecular Physiology Institute, Duke University Medical Center, Durham, NC 27710, USA

## Abstract

**Objectives:**

Dexamethasone has been shown to have analgesic properties in the general surgical population. However, the analgesic effects for women that undergo cesarean deliveries under spinal anesthesia remain unclear and may be related to the timing of dexamethasone administration. We hypothesized that intravenous dexamethasone administered before skin incision would significantly reduce postoperative opioid consumption at 24 h after cesarean delivery under spinal anesthesia with intrathecal morphine.

**Methods:**

Women undergoing elective cesarean deliveries under spinal anesthesia were randomly assigned to receive 8 mg of intravenous dexamethasone or placebo prior to skin incision. Both groups received a standardized spinal anesthetic and multimodal postoperative analgesic regime. The primary outcome was cumulative opioid consumption at 24 h. Secondary outcomes included cumulative opioid consumption at 48 h, time to first analgesic request, and pain scores at rest and on movement at 2, 24, and 48 h.

**Results:**

47 patients were analyzed—23 subjects that received dexamethasone and 24 subjects that received placebo. There was no difference in the median (Q1, Q3) cumulative opioid consumption in the first 24 hours following cesarean delivery between the dexamethasone group {15 (7.5, 20.0) mg} and the placebo group {13.75 (2.5, 31.25) mg} (*P*=0.740). There were no differences between the groups in cumulative opioid consumption at 48 h, time to first analgesic request, and pain scores.

**Conclusions:**

Intravenous dexamethasone 8 mg administered prior to skin incision did not reduce the opioid consumption of women that underwent cesarean deliveries under spinal anesthesia with intrathecal morphine and multimodal postoperative analgesic regimen.

## 1. Introduction

Inadequately controlled pain after cesarean delivery can be a significant source of morbidity for many women in the immediate postsurgical period and can increase the risk for developing chronic pain [1, 2]. Certainly, patients with poorly controlled postoperative pain may have difficulty with ambulation that can lead to complications such as atelectasis, pneumonia, and venous thromboembolism [3]. Severe pain may also interfere with maternal-infant bonding, reduce time spent breastfeeding a newborn infant, and predispose women to postpartum depression—all of which may have long-lasting consequences on the well-being of the neonate and the mother [4, 5]. However, control of postcesarean delivery pain continues to be challenging, with up to 85% of women reporting pain severe enough to interfere with daily activities such as walking, sleep, and mood in the first 24 hours of the postsurgical period [5].

Multimodal analgesic regimens for postcesarean delivery are recommended for their opioid-sparing effect and reduction in undesirable opioid related side-effects such as sedation, constipation, nausea, vomiting, and pruritus [6]. Dexamethasone, a commonly administered glucocorticoid with anti-inflammatory properties, has been used to reduce edema and tissue damage in a variety of conditions [7–9]. Studies have shown that perioperative dexamethasone, which has time to the peak effect of 45 min to 1 h, may also have analgesic efficacy in general surgical patients, particularly when administered preoperatively [10–12]. However, only a few studies have investigated the analgesic efficacy of intravenous dexamethasone in women undergoing cesarean delivery under spinal anesthesia who received intrathecal morphine with both mixed and inconclusive results [13–18]. These inconsistent findings could be partly explained by the fact that in some of these studies, analgesic efficacy was not the primary outcome, analgesic outcomes were not adequately reported, and the timing of dexamethasone administration was variable, being administered preoperatively, before skin incision, postdelivery or postoperatively. Interestingly in the 2 randomized controlled trials where dexamethasone reduced postoperative pain score, it was either administered preoperatively or following spinal anesthesia but before skin incision [13, 17]. These findings suggest that the analgesic effect of dexamethasone may be related to the timing of its administration, similar to its antiemetic effect [19, 20]. Therefore, we performed a single-center, prospective, double-blinded, randomized placebo-controlled trial to determine whether a single perioperative dose of 8 mg of intravenous (IV) dexamethasone administered before skin incision would significantly reduce postoperative opioid consumption at 24 h in women who underwent cesarean delivery under spinal anesthesia with intrathecal morphine. Our hypothesis was that a single dose of 8 mg IV dexamethasone administered before skin incision would significantly reduce postoperative opioid consumption at 24 h in women having cesarean delivery under spinal anesthesia with intrathecal morphine.

## 2. Materials and Methods

After Research Ethics Committee approval from the Duke University Health System Institutional Review Board (IRB), this prospective, double-blind, randomized, placebo-controlled trial was conducted at a single academic center from December 2014 to May 2016 (IRB No. 41334). The study was registered in http://www.ClinicalTrials.gov (identifier: NCT01812057).

Eligible study patients included English speaking, nonlaboring adult women, American Society of Anesthesiology (ASA) ≤ III, and gestational age ≥37 weeks scheduled for elective cesarean delivery under spinal or combined spinal epidural anesthesia. Women with body mass index (BMI) ≥45 kg/m^2^, diabetes mellitus (type 1, 2, and gestational), chronic hypertension, preeclampsia with or without severe features, history of intravenous drug or opioid abuse, history of chronic pain syndrome, history of opioid use in the past week, receipt of an antiemetic within 24 h prior to surgery, or non-English speaking were excluded from the study.

Eligible women were approached by the study staff to participate in the study on the day of surgery. After obtaining written informed consent, demographic information was collected. Subjects were randomly assigned to one of two groups using a computer-generated random number table in blocks of 20: dexamethasone at a dose of 8 mg IV or a normal saline placebo. The subjects' study allocation assignment was sealed in opaque envelopes, and blinded study drugs were prepared by personnel not involved in the study in identical 5 ml syringes. All women received antacid prophylaxis with 30 ml oral sodium citrate in the preoperative area. The spinal anesthetic technique was standardized, so that both groups received identical doses of intrathecal hyperbaric bupivacaine (12 mg), fentanyl (15 *μ*g), and preservative-free morphine (150 *μ*g). Dermatomal sensory block to T5 bilaterally assessed by pinprick was deemed as adequate to proceed with surgery. The study drug was administered as a slow IV bolus starting 5 minutes after the administration of spinal anesthesia by blinded personnel after the block was assessed as adequate and before skin incision. Patients with a failed block who required a repeat block or general anesthesia were withdrawn from the study.

Intraoperatively, patients received a prophylactic phenylephrine infusion for the prevention of spinal-induced hypotension as per standard protocol at our institution. A phenylephrine infusion was initiated at 50 mcg/min, titrated to maintain systolic blood pressure within 10% of baseline systolic blood pressure, and stopped 10 minutes after delivery. The baseline systolic blood pressure was determined from the mean of 3 consecutive systolic blood pressures measured in the preoperative area. Noninvasive blood pressure measurements were taken every minute until delivery and then every 2.5 minutes after delivery. If systolic blood pressures increased by more than 10% of baseline, the infusion was reduced to 25 mcg/min. If systolic blood pressure decreased by more than 10% of baseline, a bolus dose of phenylephrine 100 mcg was given. If hypotension recurred, another bolus dose of phenylephrine was given, and the infusion was doubled to 100 mcg/min. A verbal assessment of intraoperative nausea (ION) was obtained every 5 minutes for the first 15 minutes after placement of spinal block and then every 10 minutes thereafter until the end of procedure using an 11-point verbal rating scale (0 = no nausea; 10 = worst possible nausea). Intraoperative vomiting (IOV) or retching were also assessed at similar time points. If patients experienced nausea or vomiting without concurrent hypotension, they were treated first with ondansetron 4 mg IV and then metoclopramide 10 mg IV as a second agent if there was no response to ondansetron.

Acute pain in the postanesthesia care unit (PACU) was treated with IV boluses of fentanyl as needed based on a numerical rating scale (NRS) (25 mcg for NRS 4–6 and 50 mcg for NRS 7–10). The postoperative regimen was also standardized with patients receiving scheduled doses of naproxen 500 mg every 12 h and acetaminophen 975 mg every 6 h for the first 48 h beginning in PACU. Rescue opioids were administered as oral oxycodone. The dose of oxycodone was administered based on NRS for postoperative pain (oxycodone 5 mg for NRS 4–6 and 10 mg for NRS 7–10) every 4 h as needed. Rescue doses of IV morphine 2 mg were administered up to every 2 h only to patients who experienced intolerable pain not initially relieved by oral analgesia. All opioid doses were converted to IV morphine equivalents for analysis. Promethazine 6.25 mg IV was administered as a rescue antiemetic postoperatively. Nalbuphine 2.5 mg IV was administered as a rescue antipruritic. Postoperatively, wounds were assessed for signs of infection by the managing obstetric team as a part of normal clinical practice on a daily basis.

We collected data at 2 h, 24 h, and 48 h postoperatively. We assessed pain scores at rest and with movement using an 11-point NRS (0 = no pain; 10 = worst possible pain). Postoperative nausea was assessed using a similar 11-point NRS used intraoperatively. We also collected information about the incidence of vomiting and retching. Pruritus was assessed using an 11-point NRS postoperatively (0 = no pruritus; 10 = worst possible pruritus).

The primary outcome of the study was total opioid consumption at 24 h in mg morphine equivalents. Secondary outcomes included total opioid consumption at 48 h, time to first rescue analgesic request, pain scores at rest and on movement, intraoperative and postoperative nausea, vomiting, retching, need for rescue antiemetics, postoperative complete response (defined as absence of nausea, vomiting, retching, and no need for rescue antiemetics), pruritus, and the need for rescue antipruritic agents.

### 2.1. Statistical Analysis

Based on pilot data from our institution, we determined that in our patient population, a sample size of 47 study patients in each group would have 80% power to detect a difference in mean oxycodone consumption of 10 mg at 24 h (a difference between 30 mg in one group and 20 mg in the other with a common standard deviation of 17 mg) using a two-group *t*-test with a 0.05 two-sided significance level. We planned to recruit 52 patients per group to account for a 10% patient attrition rate. Demographic, patient characteristics, and outcome measures were summarized in the form of frequency (percentage) for categorical variables and means and standard deviation (SD) or median and interquartile range (IQR) for continuous variables for each treatment group. The primary outcome of total opioid consumption at 24 h was tested between treatment groups using the Wilcoxon rank-sum test. Secondary outcomes were analyzed using the chi-square or Fisher exact tests for categorical variables and the Wilcoxon rank-sum test for continuous variables as appropriate. The log-rank test was used to test the differences of Kaplan–Meier survival curves between treatment groups for time to first analgesic request. Significance level was set at 0.05.

To assess the overall impact of early termination of recruitment to this study (described below in the results), a post hoc analysis was performed to assess if the difference in the primary outcome in a smaller dataset would be similar to what we would have been able to detect if the study recruitment had been completed. In this analysis, we first resampled the total opioid consumption at 24 h from the actual sample size recruited, with replacement to generate a new dataset of 94 subjects (47 per treatment group) for 10,000 replicates. In each replicate, we then computed the mean total opioid consumption for the placebo and dexamethasone groups, respectively, along with the mean difference. The mean and 95% confidence intervals (CI) of the mean difference of the total opioid consumption at 24 h between treatment groups were computed based on these 10,000 replicates. All analyses were performed in SAS9.4 (SAS Inc. Cary, NC).

## 3. Results and Discussion

### 3.1. Results

**Figure 1:**
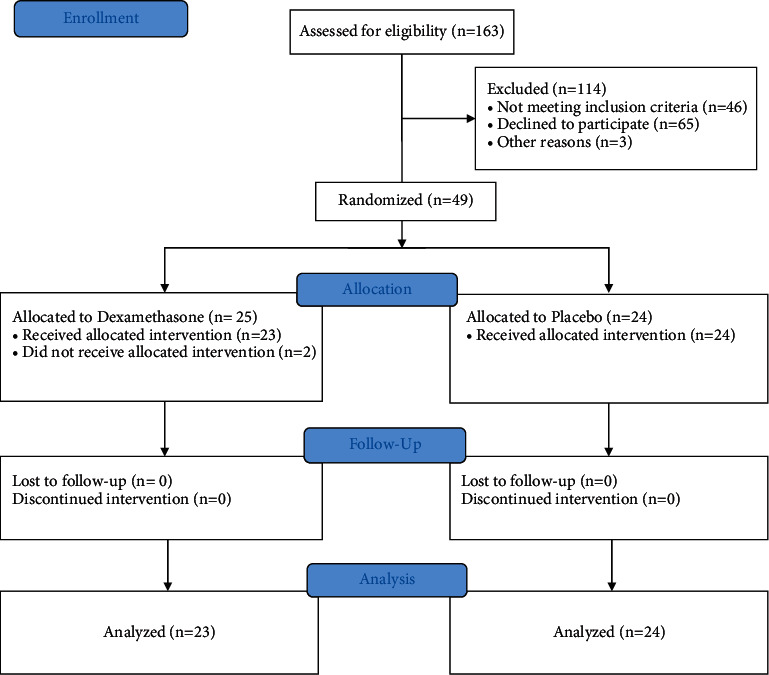
Consort 2010 flow diagram: prospective, double-blind, randomized, placebo-controlled trial of intravenous dexamethasone vs. placebo for postcesarean delivery analgesia.

We recruited patients from December 16, 2014, to May 31, 2016; the study was terminated prematurely due to a significant change in the postoperative analgesic regimen that was considered standard of care at our institution. The flow of patients is shown in Figure 1. Forty-nine patients were randomly assigned with 25 patients allocated to the dexamethasone group and 24 allocated to the placebo group. Two patients from the dexamethasone group were withdrawn before the intervention. We, therefore, analyzed data on 47 patients, 23 subjects were randomly assigned to receive dexamethasone and 24 subjects were randomly assigned to receive placebo.

Subject characteristics are given in Table 1. The groups were comparable with regards to age, BMI, American Society of Anesthesiologists' (ASA) physical status, gravidity, parity, history of previous cesarean delivery, history of ION, IOV or postoperative nausea or vomiting (PONV), or history of smoking. Intraoperative factors were also comparable between the groups.

**Figure 2:**
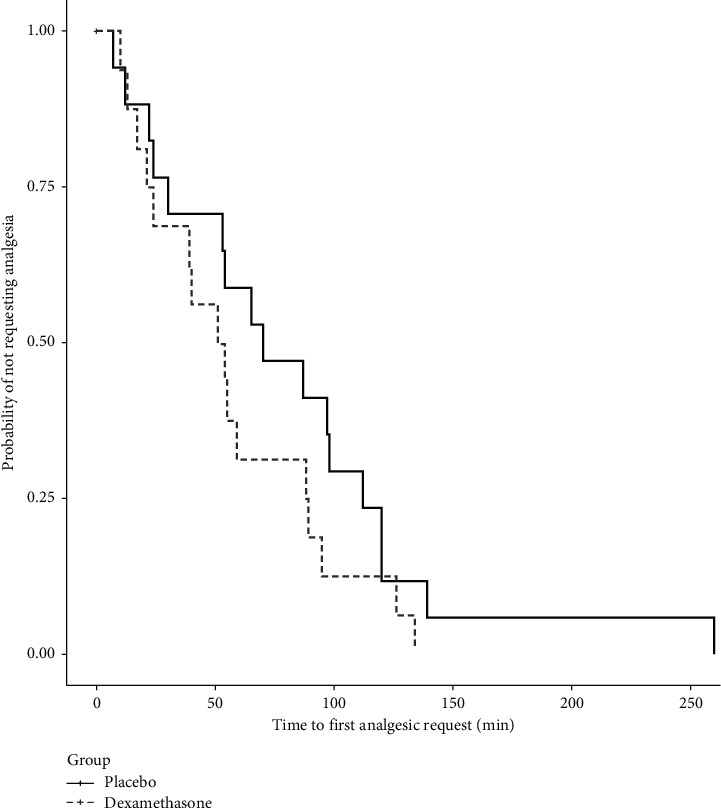
Kaplan–Meier curve for time to first analgesic request. There was no significant difference in the time to first analgesic request between cesarean delivery patients that received 8 mg dose intravenous dexamethasone intraoperatively and those that received placebo (*P*=0.196).

The postoperative analgesic outcomes are given in Table 2. The median (Q1, Q3) total opioid analgesic consumption in the first 24 h for the dexamethasone group was 15.0 (7.5, 20.0) mg morphine equivalents, and this did not significantly differ from the median (Q1, Q3) total opioid analgesic consumption in the placebo group of 13.8 (2.5, 31.2) mg morphine equivalents (*P*=0.740). Cumulative opioid consumption was also not significantly different between the groups at 48 h. There was no significant difference between the groups in the time to first analgesic request (Figure 2) or in pain scores at rest or with movement at 2, 24, and 48 h postoperatively (Table 2).

To assess if the difference in the primary outcome in our dataset was similar to what we would have been able to detect if the study had not been prematurely terminated, we performed a post hoc analysis in which we resampled the total opioid consumption at 24 h from our original 47 patients with replacement to generate a new dataset of 94 subjects (47 per treatment group) for 10,000 replicates. In the 10,000 replicates of the resampled data, the mean of the mean difference in total opioid consumption at 24 h was 3.879 mg morphine equivalents with a 95% confidence interval (CI) of −2.510 mg to 10.648 mg. This implies that even if we had successfully recruited 94 patients, we were unlikely to have detected any significant differences in the total opioid consumption at 24 h between the placebo and dexamethasone groups.

Nausea and vomiting outcomes are given in Table 3. There were no significant differences between the groups in the incidence of ION, IOV, or the need for intraoperative rescue antiemetic (Table 3). There were also no significant differences between the two groups in postoperative nausea scores or the prevalence of POV at 2, 24, and 48 h. The overall prevalence of PON was also similar between the groups (Table 3). Dexamethasone decreased the number of vomiting/retching episodes when compared with the placebo group at 2 h (*P*=0.046) (Table 3). Dexamethasone significantly reduced the need for postoperative rescue antiemetics when compared with placebo (43.5% vs. 75%, *P*=0.028) (Table 3). There were no significant differences in the incidence of postoperative pruritus and need for antipruritic agents between the groups. No patients in either the dexamethasone or placebo group developed a wound infection during hospitalization.

### 3.2. Discussion

The major finding in this study was that for women who underwent cesarean delivery under spinal anesthesia that included intrathecal morphine, a single dose of dexamethasone 8 mg IV administered prior to skin incision did not reduce postoperative analgesic consumption or pain scores. These findings are consistent with 3 recently published studies that also reported no analgesic effects of IV dexamethasone administered to women undergoing cesarean delivery with neuraxial anesthesia with intrathecal morphine [14–16]. However, dexamethasone did have an antiemetic effect as evidenced by a reduction in the number of early vomiting/retching episodes and the need for postoperative rescue antiemetics. These findings suggest that in patients that receive spinal anesthesia for cesarean delivery, a single preincisional dose of IV dexamethasone had no analgesic effect but may have some antiemetic benefits. However, these findings need to be interpreted in the context that the study was terminated prematurely and was likely underpowered for the primary outcome.

Dexamethasone has been shown to reduce postoperative pain in the general surgical population [11, 12]. Postoperative pain arises from a complex network of pathways, but a key mechanism of acute postsurgical pain arises from direct tissue disruption and subsequent regional inflammation [21–23]. The pain stimulus is thought to be caused by local tissue ischemia and edema, triggered by the release of chemomodulators such as interleukin and tumor necrosis factor and hyperalgesia from sensitization of existing pain fibers [24, 25]. Dexamethasone is a glucocorticoid with anti-inflammatory properties and multiple clinical applications. The mechanism for the analgesic properties of dexamethasone is not precisely understood but is attributed to the downregulation of prostaglandin synthesis, reduction of proinflammatory chemokines, and the altered transmission of nociception at the level of nerve tissue [26, 27].

Clinically, the analgesic effects of dexamethasone are equivocal. While two large meta-analyses demonstrated that a single dose of IV dexamethasone in the general surgical patients may have analgesic benefit, the absolute reduction in opioid consumption, pain scores, and time to first analgesic request were small and maybe of dubious clinical benefit [11, 12]. In one meta-analysis which included 45 randomized controlled trials, dexamethasone reduced the mean 2 h and 24 h opioid consumption by only 0.87 mg morphine equivalents (95% CI: −1.40, −0.33) and 2.33 mg morphine equivalents (95% CI: −4.39, −0.26), respectively, when compared with placebo [11]. Dexamethasone administration only increased the mean time to first analgesic request by 12.06 min (95% CI: 0.80, 23.32) when compared with placebo [11]. Dexamethasone administration was also only associated with a reduction in mean 2 h and 24 h pain scores of 0.49 (95% CI: −0.83, −0.15) and 0.48 (95% CI: −0.62, −0.35), respectively, when compared with placebo using an 11-point scale. A second metanalysis of 24 randomized controlled trials similarly reported only small differences in pain scores at rest and on movement (at ≤4 h and 24 h) and opioid consumption between the dexamethasone and the placebo groups in the general surgical patient population [12]. Similarly, in patients that received neuraxial morphine, including for postcesarean delivery analgesia, the administration of dexamethasone resulted in a very small reduction in pain scores at 24 h and in patients having cesarean delivery, it did not reduce the need for rescue analgesia [19]. In the postcesarean delivery patient population, the profound analgesic effect of neuraxial morphine may lead to a smaller dynamic range in postoperative opioid consumption when compared with the general surgical population. As a result, in a multimodal postcesarean analgesic regimen which includes intrathecal morphine, acetaminophen, and nonsteroidal anti-inflammatory drugs, the addition of dexamethasone may have a negligible additional analgesic benefit despite the preincisional and preemptive administration as demonstrated in the recent study by Selzer et al. [16]. One meta-analysis determined that doses of dexamethasone greater than 0.1 mg/kg reduced postoperative pain and opioid consumption [12]. In our study, we administered 8 mg which may have been just below this 0.1 mg/kg threshold and may partly explain the lack of analgesic efficacy. A prior study using a larger dose of 10 mg dexamethasone administered immediately preoperatively reported a significant reduction in the prevalence of postoperative pain especially on movement up to 24 h. However, because this study did not report opioid consumption at the relevant time points, it is difficult to determine whether this higher dose was associated with any opioid-sparing effect [13].

Dexamethasone has a longstanding role as an antiemetic. In this study, we were able to demonstrate some antiemetic efficacies in patients who received spinal anesthesia for cesarean delivery with intrathecal morphine. Intrathecal morphine administration is associated with a high incidence of PONV following cesarean delivery, and this effect is dose dependent [28, 29]. A previous meta-analysis failed to demonstrate any significant reductions in the incidence of PONV or need for rescue antiemetics with dexamethasone administration in patients receiving intrathecal morphine, possibly due to the administration of dexamethasone after rather than prior to receipt of intrathecal morphine [19]. This could partly be explained by the rapid rostral spread of preservative-free morphine following intrathecal administration exerting its emetogenic effect on the chemoreceptor trigger zone in advance of the slow onset of the antiemetic effect of intravenously administered dexamethasone [19]. In a recent study, preoperative dexamethasone administered approximately 22 minutes before the administration of intrathecal morphine and 75–80 minutes before the end of surgery did not significantly reduce the incidence of PONV [16]. However, the lack of efficacy may be related to the higher dose of 0.2 mg of intrathecal morphine used in this study by Selzer et al. [16]. In fact, with an estimated 2 h for the onset of action of the antiemetic effect of dexamethasone, preincisional administration of dexamethasone maybe a more prudent therapeutic strategy for PONV prophylaxis as has been advocated in the general surgical population, but the ideal timing needs to be further investigated [9, 20, 30].

There were significant limitations to our study. First, the early termination of the study resulted in a smaller sample size and reduced power to determine whether dexamethasone could significantly reduce 24 h opioid consumption inherently increasing the risk of a type II error. This was done because of a change in our practice and the institution of a different analgesic regimen, which was associated with significantly lower opioid consumption compared with the regimen used in this study [31]. We, therefore, felt it was unethical to continue using the older regimen for the study subjects. We applied a resampling strategy to determine the confidence interval boundaries of the mean difference of opioid consumption if we could have recruited 94 patients as originally planned. The 95% CI of the mean of mean difference of opioid consumption at 24 h included 0, which implies that even a study with the originally planned sample size would have likely yielded the same results. In this study, the difference in the mean opioid consumption between the dexamethasone and placebo groups was 3.85 mg at 24 h, a difference which would not be clinically significant. Based on this estimated mean difference, a significantly larger sample size of 284 patients per group (total *N* = 568) would be needed to detect this much smaller difference in opioid consumption at 24 h. Performing a single-center study with such a large sample size may not be feasible. Our study reported no incidence of wound infection with dexamethasone administration, but this study was also not originally powered to investigate adverse effects resulting from dexamethasone administration. Interestingly, a large meta-analysis in the general surgical population has not demonstrated an increase in infection and only a modest increase in blood glucose levels [11]. In this study, we administered a single dose of dexamethasone before surgical incision and before the delivery of the baby resulting in only short exposure of the fetus to dexamethasone. While the long-term effects of this single administration remain unclear, it is reassuring that a single course of antenatal steroids in late preterm birth infants resulted in only a small increase in neonatal hypoglycemia and no other serious adverse effects [32]. Furthermore, long-term follow-up of children born to women who received a single course of antenatal steroids at term prior to elective cesarean delivery did not reveal any adverse effects on overall health, behavior, and academic achievement when compared with those in the placebo group [33].

In summary, under the conditions of the study, dexamethasone 8 mg administered IV prior to skin incision for cesarean delivery under spinal anesthesia and in combination with a multimodal postoperative analgesic regimen that includes intrathecal morphine did not reduce maternal opioid consumption or pain scores. However, these findings need to be interpreted cautiously in light of the methodological limitations of this study. Future adequately powered studies are needed to further evaluate the analgesic efficacy of dexamethasone in women undergoing cesarean delivery under spinal anesthesia.

## Figures and Tables

**Table 1:** Subject characteristics.

	Dexamethasone (*n* = 23)	Placebo (*n* = 24)
Age (year)	32.2 (4.7)	30.63 (5.71)
Weight (kg)	90.9 (15.9)	84.9 (87.8)
BMI (kg/m^2^)	33.8 (5.9)	32.3 (5.1)
Race/ethnicity		
Caucasian	17 (73.9%)	14 (58.3%)
Black/African American	6 (26.1%)	5 (20.8%)
Asian/Indian	0 (0.0%)	3 (12.5%)
Hispanic	0 (0.0%)	2 (8.3%)
ASA status		
2	22 (95.6%)	23 (95.8%)
3	1 (4.4%)	1 (4.2%)
Gravidity	3 (2, 5)	2 (2, 4)
Parity	1 (1, 2)	1 (1, 1)
History of previous cesarean	17 (73.8%)	19 (79.2%)
History of IONV^*∗*^	4/18 (22.2%)	9/18 (50%)
History of PONV^*∗*^	5/18 (27.8%)	3/19 (15.8%)
History of smoking		
Never smoked	15 (65.2%)	15 (62.5%)
Smoked prior to pregnancy	5 (21.2%)	6 (25.0%)
Current smoker	3 (13.0%)	3 (12.5%)
Uterus exteriorized	21 (91%)	24 (100%)
Abdominal irrigation after uterine closure	23 (100%)	23 (96%)
Duration of surgery (min)	70.4 (20.4)	64.0 (22.8)
Intraoperative fluids administered (mL)	2313 (536)	2297 (545)
Dose of phenylephrine administered (mg)	2.5 (1.7, 3.2)	2.3 (1.6, 3.2)
Estimated blood loss (mL)	826 (120)	717 (140)

Mean (SD) or *n* (%); median, (Q1, Q3); IONV, intraoperative nausea and vomiting; PONV, postoperative nausea and vomiting. ^*∗*^Missing data and presented as observed count/total count per group.

**Table 2:** Postoperative analgesic outcomes.

Time	Dexamethasone (*n* = 23)	Placebo (*n* = 24)	*P* value
Total opioid consumption in morphine equivalents (mg)			
24 h	15 (7.5, 20.0)	13.8 (2.5, 31.2)	0.740
48 h	20 (10.0, 40.0)	22.5 (3.75, 48.7)	0.709
Pain score at rest			
2 h	2 (0.0, 4.0)	3.5 (1.5, 5.0)	0.190
24 h	2 (0.0, 3.0)	2.5 (1.0, 4.2)	0.267
48 h	2 (0.0, 3.0)	2 (0.0, 4.0)	0.491
Pain score with movement			
2 h	5 (2.0, 7.0)	5 (4.0, 7.0)	0.273
24 h	5 (3.0, 7.0)	5 (4.0, 6.8)	0.465
48 h	4 (3.0, 6.0)	5 (3.0, 7.0)	0.525

Median values, (Q1, Q3). *P* value is computed from the Wilcoxon rank-sum test.

**Table 3:** Intraoperative and postoperative nausea and vomiting and pruritus outcomes.

	Dexamethasone (*n* = 23)	Placebo (*n* = 24)	*P* value
Incidence of intraoperative nausea	5 (21.7%)	2 (8.3%)	0.245^2^
Incidence of intraoperative vomiting	4 (17.4%)	5 (20.8%)	1.000^2^
Intraoperative antiemetic administered	10 (43.5%)	9 (37.5%)	0.676^3^
Postoperative nausea scores			
2 h	0 (0, 4)	1.5 (0, 8.5)	0.058^1^
24 h	0 (0, 0)	0 (0, 4.5)	0.149^1^
48 h	0 (0, 0)	0 (0, 0)	0.563^1^
Incidence of postoperative nausea	10 (43.5%)	15 (62.5%)	0.191^3^
Incidence of postoperative vomiting/retching			
2 h	4 (17.4%)	11 (45.8%)	0.060^2^
24 h	4 (17.4%)	10 (41.6%)	0.111^2^
48 h	0	0	
Number of vomiting/retching episodes			
2 h	0 (0, 0)	0 (0, 2.0)	0.046^1^
24 h	0 (0, 0)	0 (0, 2.0)	0.065^1^
48 h	0 (0, 0)	0 (0, 0)	
Postoperative antiemetic administered	10 (43.5%)	18 (75.0%)	0.028^3^
Complete response	1 (4.3%)	2 (8.3%)	1.000^2^
Postoperative pruritus	16 (69.5%)	17 (70.8%)	0.924^3^
Postoperative antipruritic administered	12 (52%)	12 (50%)	0.881^3^

*N* (%); median values (Q1, Q3); IONV (intraoperative nausea and vomiting); PONV (postoperative nausea and vomiting). ^1^Wilcoxon rank-sum test. ^2^Fisher exact test. ^3^Chi-square test.

## Data Availability

The data that support the findings of this study are available from the corresponding author upon reasonable request.

## Additional Points

*Main Points*. Postcesarean delivery analgesia after spinal anesthesia still remains a challenge. This randomized controlled trial investigated the analgesic efficacy of dexamethasone. Dexamethasone 8 mg did not reduce 24 h opioid consumption after cesarean delivery. Dexamethasone did not reduce pain scores at 2, 24, and 48 h after cesarean delivery.
